# Case Report: Euglycemic diabetic ketoacidosis as the initial presentation of adult-onset autoimmune diabetes in a treatment-naïve man

**DOI:** 10.3389/fendo.2026.1996912

**Published:** 2026-10-30

**Authors:** Sai Prasad, Anukriti Sharma, Anubhooti Sharma, Nishita Malu, Tanisha Jindal, Parth Shah, Angshuman Borpatra Gohain, Dileep Raja Kuraku

**Affiliations:** 1 Department of Internal Medicine, S. Nijalingappa Medical College, Bagalkot, Karnataka, India; 2 Department of Internal Medicine, Jaipur National University Institute of Medical Sciences and Research Centre, Jaipur, Rajasthan, India; 3 Department of Internal Medicine, Topiwala National Medical College, Mumbai, Maharashtra, India; 4 Department of Internal Medicine, MP Shah Government Medical College, Jamnagar, Gujarat, India; 5 Department of Internal Medicine, Lakhimpur Medical College, Chowkham, Assam, India; 6 Department of Internal Medicine, National Pirogov Memorial Medical University, Vinnytsia, Ukraine

**Keywords:** adult-onset autoimmune diabetes, case report, C-peptide, euglycemic diabetic ketoacidosis, islet autoantibodies, latent autoimmune diabetes in adults, type 1 diabetes

## Abstract

**Background:**

Autoimmune diabetes can begin in adulthood and is often initially mistaken for type 2 diabetes. Its first presentation as euglycemic diabetic ketoacidosis (EDKA), that is, ketoacidosis with a plasma glucose below 200 mg/dL (11.1 mmol/L), is uncommon and easily overlooked, particularly in a patient without known diabetes or sodium-glucose cotransporter-2 (SGLT2) inhibitor exposure.

**Case presentation:**

A 34-year-old previously healthy, lean man (body mass index 21.4 kg/m²) presented with a three-day history of fatigue, nausea, reduced appetite, increased thirst, and mild dyspnea, with oral intake falling to approximately 700 kcal on the day before admission. He had a high anion gap metabolic acidosis (pH 7.23, bicarbonate 13 mmol/L, anion gap 22 mmol/L) with marked ketonemia (β-hydroxybutyrate 5.2 mmol/L), but a random blood glucose of only 198 mg/dL (10.99 mmol/L). He had no known diabetes, took no medications, and did not drink alcohol. Diabetes was established during the admission by a hemoglobin A1c of 7.1%, and positivity for GAD65, IA-2, and ZnT8 antibodies with a low fasting C-peptide (0.42 ng/mL) indicated adult-onset autoimmune diabetes. Infectious, alcoholic, toxicologic, lactic, renal, and adrenal causes were excluded, and the brief reduction in intake was insufficient to explain the severity of the ketoacidosis. The episode resolved within 24 hours with intravenous fluids, insulin infusion with concurrent dextrose, and potassium replacement. He was discharged on basal–bolus insulin and remained free of ketoacidosis at three months, with a persistently low C-peptide.

**Conclusion:**

Clinically significant ketoacidosis can precede the diagnosis of diabetes and occur with a glucose below the conventional threshold. An unexplained high anion gap acidosis should prompt ketone measurement regardless of the glucose level or a history of diabetes. Whether this patient has latent autoimmune diabetes in adults (LADA) or adult-onset type 1 diabetes will require longer follow-up to determine.

## Introduction

Autoimmune diabetes is not confined to childhood. A substantial proportion of cases begin in adulthood, where they are frequently misclassified as type 2 diabetes at diagnosis (1, 2). This group includes classic adult-onset type 1 diabetes and a more slowly progressive phenotype, latent autoimmune diabetes in adults (LADA), which shares features of both type 1 and type 2 diabetes (3) and accounts for approximately 2–12% of adults initially diagnosed with type 2 diabetes, including Indian patients (4, 5). LADA is usually defined by onset at 30 years of age or older, the presence of islet autoantibodies, and independence from insulin for at least the first six months after diagnosis, although whether it is a distinct entity or part of a continuum with adult-onset type 1 diabetes remains debated (6–10).

Although diabetic ketoacidosis (DKA) has traditionally been associated with marked hyperglycemia (11), the 2024 international consensus report defines euglycemic DKA (EDKA) as DKA with a plasma glucose below 200 mg/dL (11.1 mmol/L) (12). EDKA has been described in pregnancy, starvation, and heavy alcohol use and, most prominently, with sodium-glucose cotransporter-2 (SGLT2) inhibitor therapy (13–16). Because a glucose below 200 mg/dL satisfies the consensus criteria only when diabetes is already known, ketoacidosis with near-normal glucose is a particular diagnostic challenge in a patient without previously diagnosed diabetes or SGLT2 inhibitor exposure, in whom the illness may be attributed to gastroenteritis or another non-diabetic cause and recognition delayed (16, 17).

This case is worth reporting because ketoacidosis with a glucose below 200 mg/dL was the first manifestation of adult-onset autoimmune diabetes in a treatment-naïve adult without SGLT2 inhibitor exposure or prolonged fasting. It shows how such an episode can fall outside the formal DKA criteria at presentation, and why recognition should rest on the acid–base findings and ketone measurement rather than on the glucose level. The report was prepared in accordance with the CARE guidelines (18).

## Case presentation

### Patient information

A 34-year-old man with no prior medical history presented to the emergency department with a three-day history of progressively worsening generalized fatigue, persistent nausea, and decreased appetite. Over the same period, he had noticed increased thirst and mild breathlessness at rest. He reported no polyuria, no significant unintentional weight loss, and no recent illness. He was not taking SGLT2 inhibitors, corticosteroids, or any other medication, did not consume alcohol, and had not been fasting. The family history was negative for diabetes mellitus.

Because reduced intake is a recognized contributor to EDKA, a structured dietary recall was obtained for the 72 hours before admission. His estimated intake was approximately 1700 kcal on day −3, 1200 kcal on day −2 as his appetite fell, and 700 kcal on day −1, taken mainly as liquids, with an estimated carbohydrate intake of 60–80 g over the final 24 hours. He vomited once, on the day of presentation. This represented a modest, short-lived reduction in intake rather than prolonged fasting or starvation.

### Clinical findings

On examination, he was alert, fully oriented, and not in acute distress. His heart rate was 98 beats per minute and respiratory rate 20 breaths per minute; blood pressure and temperature were within normal limits, and Kussmaul respiration was not observed. His height was 175 cm and weight 65.5 kg (body mass index 21.4 kg/m²), and his waist circumference was 78 cm, with no features suggestive of insulin resistance or metabolic syndrome. There were no clinical signs of dehydration, and the cardiopulmonary, abdominal, and neurological examinations were unremarkable.

### Timeline

The clinical course from symptom onset to the three-month review is shown in **Figure 1**, and serial metabolic values recorded during acute treatment are presented in **Table 1**.

**Figure 1:**
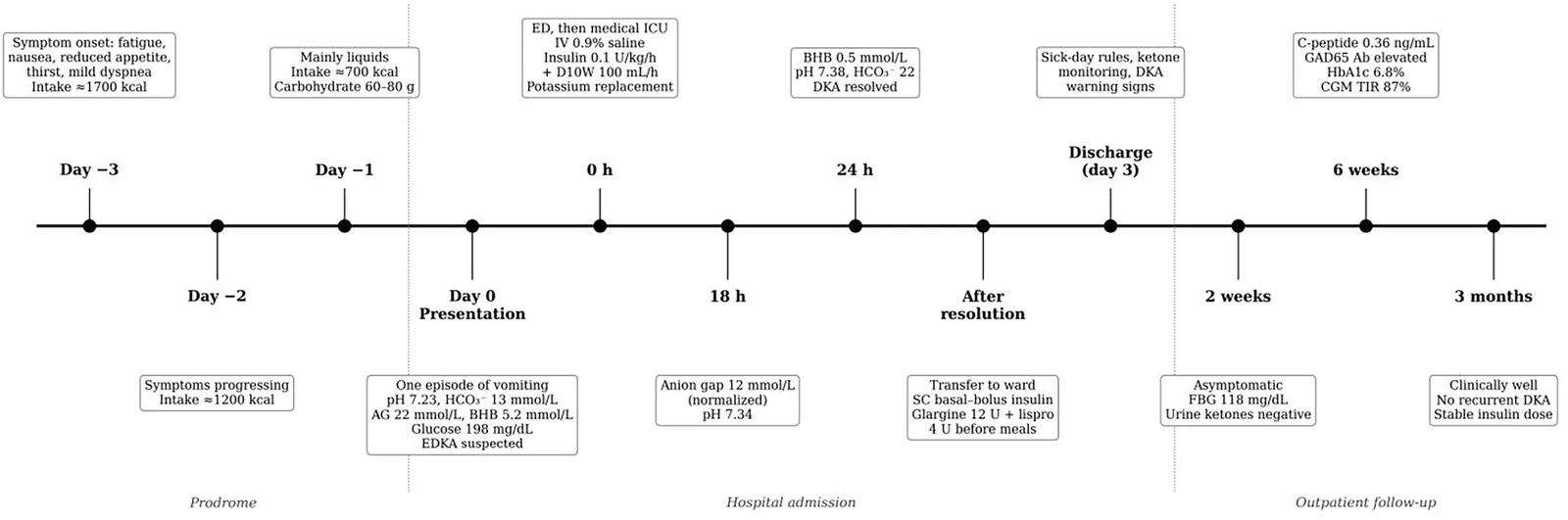
Clinical timeline. Graphical timeline of the clinical course, from symptom onset through presentation, acute treatment, and follow-up to three months. ED, emergency department; ICU, intensive care unit; D10W, 10% dextrose in water; AG, anion gap; BHB, β-hydroxybutyrate; HCO_3_^-^, bicarbonate; EDKA, euglycemic diabetic ketoacidosis; DKA, diabetic ketoacidosis; IV, intravenous; SC, subcutaneous; FBG, fasting blood glucose; GAD65 Ab, glutamic acid decarboxylase 65 antibody; HbA1c, hemoglobin A1c; CGM, continuous glucose monitoring; TIR, time in range (70–180 mg/dL).

**Table 1:** Serial metabolic parameters during acute treatment.

Parameter	0 h	6 h	12 h	18 h	24 h
Blood glucose (mg/dL)	198	154	142	136	128
Arterial pH	7.23	7.27	7.30	7.34	7.38
Serum bicarbonate (mmol/L)	13	16	18	20	22
Anion gap (mmol/L)	22	18	14	12	10
Serum β-hydroxybutyrate (mmol/L)	5.2	3.4	1.8	0.9	0.5
Serum potassium (mmol/L)	4.3	3.9	4.2	4.1	4.0

Time points refer to hours from initiation of insulin infusion. Arterial blood gases were obtained every 4 hours and β-hydroxybutyrate every 6 hours; values shown are those recorded closest to each time point. Anion gap was calculated as sodium − (chloride + bicarbonate); reference range 8–12 mmol/L. Dextrose (D10W) was infused from 0 hours.

### Diagnostic assessment

The principal laboratory findings are summarized in **Table 2**. Initial blood tests showed a high anion gap metabolic acidosis (arterial pH 7.23, serum bicarbonate 13 mmol/L, anion gap 22 mmol/L), whereas the random blood glucose was only 198 mg/dL (10.99 mmol/L). Because the glucose did not suggest a hyperglycemic crisis, the evaluation was guided by the unexplained anion gap rather than by the glucose value. Ketones were measured as part of this evaluation in a patient with nausea, thirst, and reduced intake, and showed marked ketonemia (serum β-hydroxybutyrate 5.2 mmol/L; urine ketones 3+). Serum lactate, creatinine, and osmolality were within their reference ranges, which identified ketoacidosis as the cause of the anion gap.

**Table 2:** Key laboratory findings at presentation and follow-up.

Parameter	Result at presentation	Reference range	Follow-up result (time point)
Biochemical evidence of ketoacidosis at presentation			
Arterial pH	7.23	7.35–7.45	—
Serum bicarbonate	13 mmol/L	22–26 mmol/L	—
Anion gap	22 mmol/L	8–12 mmol/L	—
Serum β-hydroxybutyrate	5.2 mmol/L	< 0.6 mmol/L	Normal at 6 weeks
Urine ketones	Large (3+)	Negative	Negative at 2 and 6 weeks
Random blood glucose	198 mg/dL (10.99 mmol/L)	70–140 mg/dL (3.9–7.8 mmol/L)	—
Evidence establishing diabetes			
Hemoglobin A1c	7.1% (54 mmol/mol)	< 5.7% (< 39 mmol/mol)	6.8% (51 mmol/mol) at 6 weeks
Fasting blood glucose	—	70–100 mg/dL (3.9–5.6 mmol/L)	118 mg/dL (6.6 mmol/L) at 2 weeks, on insulin
Evidence supporting autoimmune etiology			
GAD65 antibody	Positive (high titer)	Negative	Positive (high titer) at 6 weeks and 3 months
IA-2 antibody	Positive	Negative	—
ZnT8 antibody	Positive	Negative	—
Evidence of persistent insulin deficiency			
Fasting C-peptide	0.42 ng/mL (0.14 nmol/L)	0.8–3.1 ng/mL (0.26–1.03 nmol/L)	0.36 ng/mL (0.12 nmol/L) at 6 weeks
Associated autoimmune findings			
Anti-TPO antibody	186 IU/mL	< 35 IU/mL	—
Anti-thyroglobulin antibody	42 IU/mL	< 40 IU/mL	—
TSH	2.3 mIU/L	Within laboratory reference range	—
Free T4	1.2 ng/dL	Within laboratory reference range	—
Tissue transglutaminase IgA	3 U/mL	< 20 U/mL	—
Endomysial antibody	Negative	Negative	—

HbA1c, hemoglobin A1c; GAD65, glutamic acid decarboxylase 65; IA-2, insulinoma-associated protein 2; ZnT8, zinc transporter 8; TPO, thyroid peroxidase; TSH, thyroid-stimulating hormone; T4, thyroxine; IgA, immunoglobulin A. SI units are shown in parentheses where applicable. The time point of each follow-up measurement is stated in the final column; — indicates not repeated. Anion gap was calculated as sodium − (chloride + bicarbonate). Total IgA was normal. Other investigations at presentation were within reference ranges: serum sodium 140 mmol/L, chloride 105 mmol/L, potassium 4.3 mmol/L, lactate 0.8 mmol/L, creatinine 0.9 mg/dL (79.6 μmol/L), osmolality 280 mOsm/kg H_2_O, and morning (8 AM) serum cortisol 18.6 µg/dL; blood ethanol and the urine toxicology screen (amphetamines, cocaine, opioids, cannabinoids, and benzodiazepines) were negative.

At presentation, the biochemical criteria for DKA were not fulfilled. The ketone and acidosis criteria of the 2024 consensus report were met, but the glucose was below 200 mg/dL and the patient had no known diabetes, so the glucose criterion was not satisfied (12). The working diagnosis was therefore ketoacidosis with suspected EDKA, and treatment was started on that basis. Diabetes was established later in the same admission by a hemoglobin A1c (HbA1c) of 7.1% (54 mmol/mol) and was confirmed by an HbA1c of 6.8% (51 mmol/mol) at six weeks (2). Only once diabetes had been established could the episode be classified, in retrospect, as DKA of moderate severity (pH 7.00–7.24, bicarbonate 10 to <15 mmol/L) with a glucose below 200 mg/dL, that is, EDKA (12).

To establish the cause, islet autoantibodies were measured. GAD65 antibodies were positive at a high titer, and IA-2 and ZnT8 antibodies were also positive. Fasting C-peptide was low at 0.42 ng/mL (0.14 nmol/L). Because a value obtained shortly after ketoacidosis may underestimate residual β-cell function (19), it was repeated at six weeks and remained low at 0.36 ng/mL (0.12 nmol/L), indicating persistent insulin deficiency. Given the recognized clustering of organ-specific autoimmunity with autoimmune diabetes (20), thyroid and celiac screening was also performed. Anti-thyroid peroxidase (TPO) antibodies were clearly positive and anti-thyroglobulin antibodies marginally positive, with normal thyroid-stimulating hormone (TSH) and free thyroxine (T4) levels, indicating thyroid autoimmunity without thyroid dysfunction. Celiac serology was negative.

Infectious, alcoholic, toxicologic, lactic, renal, and adrenal causes of ketoacidosis were considered, and the relevant investigations were unrevealing. A complete blood count with differential, C-reactive protein, urinalysis, urine and blood cultures, and chest radiography showed no evidence of infection; serum lactate, creatinine, and osmolality were normal; blood ethanol and a urine toxicology screen were negative; and a morning serum cortisol of 18.6 µg/dL made adrenal insufficiency unlikely.

Starvation ketoacidosis was the most important alternative diagnosis, because his intake had fallen over the preceding three days. Starvation ketosis usually follows prolonged fasting and causes only mild acidosis, with serum bicarbonate rarely below 18 mmol/L (11). In this patient, the reduction in intake lasted only three days, he still took approximately 700 kcal and 60–80 g of carbohydrate on the final day, and the acidosis (bicarbonate 13 mmol/L) and ketonemia (β-hydroxybutyrate 5.2 mmol/L) were more severe than this degree of reduced intake would be expected to produce. The persistently low C-peptide at six weeks, when he was eating normally, confirmed underlying insulin deficiency. Reduced carbohydrate intake nevertheless probably contributed to the euglycemic phenotype by limiting glycogen stores and hepatic glucose output (16, 17), even though its duration and severity were insufficient to explain the ketoacidosis independently. The episode was therefore attributed to newly presenting insulin-deficient autoimmune diabetes, with reduced intake as a contributor to the near-normal glucose.

Positivity for three islet autoantibodies together with a persistently low C-peptide strongly supports autoimmune diabetes, and the condition was therefore classified as adult-onset autoimmune diabetes. Although the patient met the age and autoantibody components of the operational LADA definition, the LADA phenotype could not be established, because ketoacidosis made immediate insulin therapy necessary and the insulin-independence criterion could not be assessed (8).

### Therapeutic intervention and clinical course

Treatment was started in the emergency department, and the patient was admitted to the medical intensive care unit for insulin infusion and close metabolic monitoring. Management followed a standard DKA protocol consistent with current consensus recommendations (12, 21). Intravenous 0.9% sodium chloride was given at 1 L over the first hour and then at 250 mL/h, and a fixed-rate intravenous regular insulin infusion was started at 0.1 units/kg/h. Because the glucose was already below 250 mg/dL, 10% dextrose in water (D10W) at 100 mL/h was started together with the insulin infusion, so that insulin could be continued at a dose sufficient to suppress ketogenesis without causing hypoglycemia; the dextrose infusion was then adjusted according to serial glucose measurements. The serum potassium was 4.3 mmol/L at presentation, and potassium was supplemented as required to a target of 4.0–5.0 mmol/L. Arterial blood gases were repeated every four hours and serum β-hydroxybutyrate every six hours.

The serial values are shown in **Table 1**. Over the first 24 hours, the pH rose from 7.23 to 7.38, bicarbonate from 13 to 22 mmol/L, and serum β-hydroxybutyrate fell from 5.2 to 0.5 mmol/L, while glucose remained between 128 and 154 mg/dL on dextrose. The anion gap had normalized (12 mmol/L) by 18 hours, and by 24 hours the ketoacidosis had resolved according to the consensus criteria (12). Potassium remained between 3.9 and 4.3 mmol/L, and no hypoglycemia, clinically significant hypokalemia, or other treatment-related adverse event occurred. He was then transferred to the general medical ward. Once he was tolerating oral intake, he was started on subcutaneous insulin glargine 12 units at bedtime and insulin lispro 4 units before each meal, and the intravenous insulin infusion was discontinued. Before discharge, he received education on insulin administration, sick-day rules, home ketone monitoring, and the warning signs of recurrent DKA. He was discharged home on hospital day 3.

### Follow-up and outcomes

At the two-week review, he was asymptomatic, with a fasting blood glucose of 118 mg/dL (6.6 mmol/L) and negative urine ketones. Insulin doses were titrated using self-monitoring of blood glucose, and continuous glucose monitoring (CGM) was started once he was clinically stable. At six weeks, fasting C-peptide remained low at 0.36 ng/mL (0.12 nmol/L), GAD65 antibody titers remained elevated, HbA1c had fallen to 6.8% (51 mmol/mol), and CGM showed 87% of readings within 70–180 mg/dL; the basal–bolus regimen was continued unchanged. At three months, he was clinically well, with no further episodes of ketoacidosis and stable insulin requirements. C-peptide and insulin requirements will be reassessed at future visits, and periodic thyroid function testing has been planned in view of his thyroid antibody positivity.

## Discussion

This case illustrates a diagnostic gap in which clinically significant ketoacidosis occurs before diabetes has been recognized and with a glucose below the conventional threshold. The 2024 consensus report lowered the glucose threshold for DKA and allows a prior history of diabetes to satisfy the glucose criterion irrespective of the glucose value (12). This accommodates EDKA in people with known diabetes, but not in a person whose first episode of ketoacidosis is also the first sign of diabetes. In our patient, the ketosis and acidosis criteria were met at presentation, yet the episode could be classified as DKA only after HbA1c had established diabetes. A clinician who relies on glucose to screen for DKA could easily have attributed his nausea and malaise to gastroenteritis (16, 17); recognition depended instead on interpreting the high anion gap acidosis and measuring ketones. We consider this the principal educational point of the case.

In the published reports we identified, which are illustrative rather than exhaustive, EDKA or DKA in adults with autoimmune diabetes occurred mainly in patients with established diabetes, following SGLT2 inhibitor exposure (22, 23), after prolonged reduced oral intake (24), or during acute infection (25). DKA was the first presentation of LADA in one report, but with marked hyperglycemia and concurrent hashitoxicosis (26). Our patient differed in having no prior diagnosis of diabetes, no exposure to glucose-lowering drugs, a glucose in the euglycemic range, and only a short, modest reduction in intake.

The euglycemic pattern is best explained by the balance between insulin deficiency and glucose supply. Autoimmune β-cell loss reduced insulin secretion enough to release lipolysis and hepatic ketogenesis from suppression, while residual insulin secretion and several days of reduced carbohydrate intake limited hepatic glucose output, so that ketogenesis proceeded without marked hyperglycemia (17, 27). This differs from SGLT2 inhibitor-associated EDKA, in which glycosuria lowers the plasma glucose and the resulting fall in insulin and rise in glucagon favor ketogenesis (15).

The patient cannot yet be classified definitively as having LADA. The operational definition requires independence from insulin for at least six months after diagnosis, a criterion that cannot be applied when the disease presents with ketoacidosis and insulin is needed from the outset (8). Moreover, positivity for multiple autoantibodies and a low C-peptide at diagnosis are more typical of adult-onset type 1 diabetes and have been associated with faster β-cell decline than isolated GAD65 positivity (7, 28). Some authors regard LADA as part of a continuous spectrum of autoimmune diabetes rather than a separate entity (3, 6, 9). Near-term management is the same whichever label is ultimately applied, but the antibody panel remained useful: it established the etiology, informed prognostic counseling, reinforced the need to avoid SGLT2 inhibitors (8, 15), and prompted screening that identified thyroid autoimmunity (10, 20).

Several lessons follow from this case. First, an unexplained high anion gap metabolic acidosis should prompt measurement of blood β-hydroxybutyrate, and DKA should be considered regardless of the glucose level and whether or not diabetes is known (12). Second, a glucose just below 200 mg/dL does not exclude a diabetic cause, and HbA1c should be measured early to establish whether diabetes is present. Third, starvation ketosis should not be accepted as the explanation when the acidosis is more severe than the degree of reduced intake would predict, although reduced intake may still contribute to a euglycemic presentation. Finally, when ketoacidosis reveals new diabetes in a lean adult, measurement of islet autoantibodies and C-peptide clarifies the etiology and guides long-term management (10, 19).

This report has limitations. It describes a single patient, so the findings cannot be generalized. The duration of follow-up is short, and three months is insufficient to distinguish LADA from adult-onset type 1 diabetes definitively; this distinction will depend on the course of C-peptide and insulin requirements over time. The strengths of the report include the comprehensive autoimmune evaluation, the systematic assessment of alternative causes of ketoacidosis, and the structured dietary recall, which allowed the contribution of reduced intake to be assessed rather than assumed.

## Patient perspective

“I initially thought my symptoms were due to a temporary illness because I had never been diagnosed with diabetes and had generally been healthy. I was surprised to learn that I had autoimmune diabetes and that it had caused ketoacidosis even though my blood sugar was not very high. Starting insulin was initially overwhelming, but after the doctors explained why I needed it and taught me how to administer it and monitor my glucose and ketones, I became more comfortable managing the condition. I am grateful that the diagnosis was recognized and that I now understand the warning signs that should prompt me to seek medical attention.”

## Conclusion

EDKA can be the first manifestation of adult-onset autoimmune diabetes, occurring before diabetes is recognized and without SGLT2 inhibitor exposure. Because such an episode may not meet the formal DKA criteria at presentation, clinicians should measure ketones in any unexplained high anion gap acidosis rather than rely on the glucose level. Islet autoantibodies and C-peptide then clarify the etiology, whereas the distinction between LADA and adult-onset type 1 diabetes requires longer follow-up.

## Data Availability

The original contributions presented in the study are included in the article/supplementary material. Further inquiries can be directed to the corresponding author.
